# Exploring the role of the human microbiome in forensic identification: opportunities and challenges

**DOI:** 10.1007/s00414-024-03217-z

**Published:** 2024-04-10

**Authors:** Lorenzo Franceschetti, Giorgia Lodetti, Alberto Blandino, Alberto Amadasi, Valentina Bugelli

**Affiliations:** 1 Institute of Legal Medicine, Department of Biomedical Sciences for Health, University of Milan, via Luigi Mangiagalli 37, Milan, 20133 Italy; 2 Vita-Salute San Raffaele University, Milan, Italy; 3 Institute of Legal Medicine and Forensic Sciences, University Medical Centre Charité, University of Berlin, Turmstr. 21, Building N, Berlin, 10559 Germany; 4 Department of Medicine and Surgery, Section of Forensic Medicine, University of Parma, Parma, Italy

**Keywords:** Forensic microbiology, Human microbiome, Forensic identification, Microbiota profiling, Personal identification

## Abstract

Forensic microbiology is rapidly emerging as a novel tool for human identification. The human microbiome, comprising diverse microbial communities including fungi, bacteria, protozoa, and viruses, is unique to each individual, offering a new dimension to forensic investigations. While traditional identification methods primarily rely on DNA profiling and fingerprint analysis, they face limitations when complete DNA or fingerprints profiles are unattainable or degraded. In this context, the microbial signatures of the human skin microbiome present a promising alternative due to their resilience to environmental stresses and individual-specific composition. This review explores the potential of microbiome analysis in forensic human identification, evaluating its applications, advantages, limitations, and future prospects. The uniqueness of an individual’s microbial community, particularly the skin microbiota, can provide distinctive biological markers for identification purposes, while technological advancements like 16 S rRNA sequencing and metagenomic shotgun sequencing are enhancing the specificity of microbial identification, enabling detailed analysis of these complex ecological communities. Despite these promising findings, current research has not yet achieved a level of identification probability that could establish microbial analysis as a stand-alone evidence tool. Therefore, it is presently considered ancillary to traditional methods, contributing to a more comprehensive biological profile of individuals.

## Introduction

Human microbiome, as Lederberg coined, represents the collection of genome sequences from “the ecological community of commensal, symbiotic, and pathogenic microorganisms that share our body space”, including fungi, bacteria, protozoa, and viruses, that compose the microbiota [1].

One of the major potential advantages of microbiome analysis used in human forensic identification could be the uniqueness of the microbial community in each person. According to several studies, the human microbiome consists of 10–100 trillion symbiotic microbial cells unique to each individual. Moreover, in a reference man (age 20–30 years; weight 70 kg, height 170 cm) the count is estimated to be 3.8 × 10^13^ cells, with a total mass of 0.2 kg [2, 3]. These organisms are distributed through the different anatomical sites, according to which they present a specific taxonomic composition. A taxon refers to any group or rank in a biological classification, such as a phylum, order, family, genus, or species, into which related organisms are classified.

Despite considerable interpersonal variability, the core microbiome represents a collection of bacterial communities shared within individuals, for example Propionibacterium acnes, a commensal of human skin.

The relationship between humans and their microbiome offers a reservoir of information, that could be useful for identification. Since human identification plays a primary role in forensic for many legal reasons, including criminal matters such as guilt and impersonation, civil issues, such as inheritance or reunification of orphaned children with other relatives, administrative, ethical, and humanitarian reasons [4], the present review aims at updating the microbiome study in forensic human identification, shedding light on how forensic microbiology entities are reshaping the landscape of forensic investigations. The purpose is also to focus on its applications, benefits, limitations, and future perspectives, in order to understand the robustness and reliability of such studies, and their applications in Court. Moreover, this paper can serve as a valuable resource for forensic practitioners confronted with the challenge of identifying unknown individuals using forensic microbiology techniques, particularly in cases where other methods cannot be used.

## Materials and methods

### Eligibility criteria

This systematic review was conducted in adherence to the guidelines stipulated by the Preferred Reporting Items for Systematic Reviews and Meta-Analyses (PRISMA) [5, 6].

### Search criteria and critical appraisal

A comprehensive review of literature and a thorough evaluation of the gathered studies were undertaken. The databases PubMed, Science Direct Scopus, and Excerpta Medica Database (EMBASE) were utilized to carry out the analysis, spanning from their establishment to October 2023.

The following query was used:

*(“forensic microbiology” OR “microbial forensics” OR “forensic microbial analysis” OR “microbiological evidence”) AND (“human identification” OR “biological identification” OR “identity determination” OR “forensic identification”)*.

Results were then filtered for publications in English, resulting in 35 publications. For each paper included in the literature review, the title, authors, journal, year, and type of publication were extracted. Bibliographies of all identified papers were reviewed and compared to identify additional relevant literature. Methodological evaluation of each study was conducted according to PRISMA standards, including assessment of bias. All researchers independently reviewed the papers for which the title or abstract appeared relevant.

Disagreements on eligibility among researchers were resolved by a consensus process. All researchers independently reviewed papers for which the title or abstract appeared relevant and selected those that analyzed microbiome with “human identification”.

**Fig. 1:**
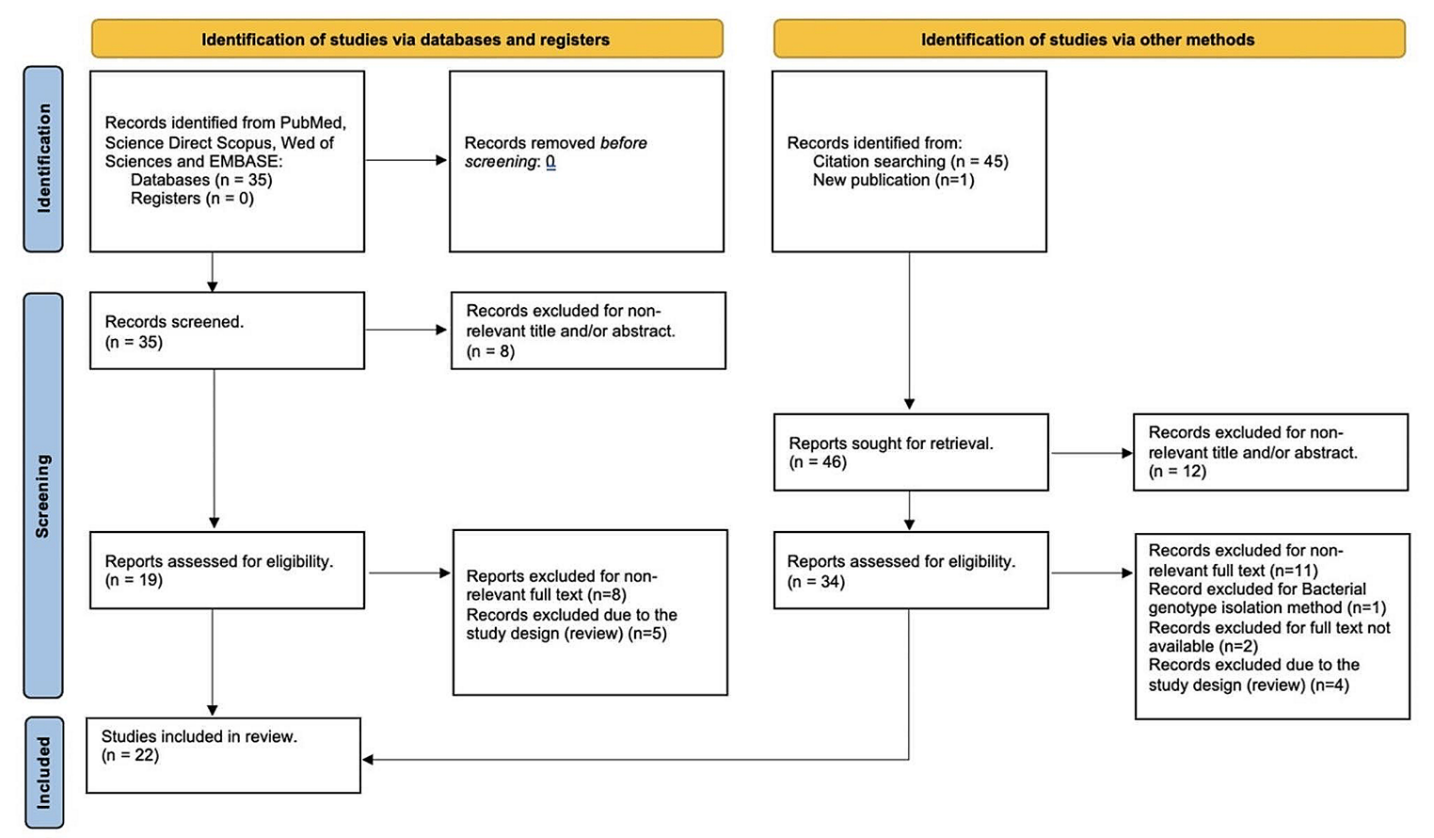
PRISMA 2020 flow diagram for new systematic reviews which included searches of databases, registers, and other sources

In the screening phase, publications clearly falling out of scope with respect to the aim of this review were excluded. After the screening phase, 19 publications were assessed as eligible for full-text assessment. Finally, 46 articles were added through backward search (analyzing the cited references in the selected articles), resulting in further 34 articles eligible for full-text assessment. Finally, 22 articles were included in the systematic review. Figure 1 shows the PRISMA chart which synthetically describes the screening and the inclusion process of the selected articles.

### Risk of bias

Highlights of this systematic review include number and breadth of the collected studies, which span the globe; the hand search and scan of reference lists for the identification of all relevant studies; and a flowchart that describe in detail the study selection process. Despite our efforts to fairly evaluate the existing literature, this review includes studies that were published in a time frame of few decades; thus, these results should be interpreted considering that the accuracy of scientific procedures may change over the years, especially in the field of molecular biology.

## Results and discussion

Twenty-two papers dealing with microbiome and human identification that fulfilled the inclusion criteria were included in the investigation [8–29]. The main characteristics of the articles including authors, year, reference number, sample, main findings, and limitations, are comprehensively reported in Table 1. No case reports or reviews were selected.

**Table 1:** Summary of the 22 selected articles. This table presents a comprehensive overview, listing the authors, publication year, and reference number, alongside the study sample, main findings, and noted limitations

Authors, Year, [Ref]	Study Sample	Main Findings	Limitations
Fierer et al., 2010 [8]	-For the keyboard study, three pc and their owners, 15 other private and public pc -For the computer mice study, 9 individuals and 270 other hands sampled	-Greater similarity between personal objects and owners (significantly for the computer mice study) -No significantly influence on storage under typical indoor conditions for up to 14 days	-Sample size -Individuals shared office space/same buildings -Sample and storage conditions far from reality (for example control environmental)
Schmedes et al., Sept. 2017 [9]	-14 skin body sites from 12 individuals sampled at 3 time points over a > 2.5-year period	-The manubrium site and the hypothenar palm yield highly accurate rates of classification (97% and 96% respectively). Nucleotide diversity of stable markers yielded accuracies as high as 100% from some body parts and contributed significantly greater to classification accuracies (*p* < 0.01).	-Sample size -Low intraindividual samples -Uncertain applicability to real forensic applications
Schmedes et al., Oct. 2017 [10]	-8 individuals, swabs from 3 body sites (foot, hand and manubrium): 3 replicate samples collected from each body site ◊ *N* = 72	-Classification accuracies were highest for Hand (average 97.9% SD 2.1) than for Manubrium (average 86.3% SD 6.9) using universal markers. Their accuracy calculated using enriched markers were comparable (*p* = 1). -Classification accuracies for foot were significantly higher (*p* < 0.00001) using enriched markers (up to 92%) than universal markers (up to 23%). Body site origin was predicted with up to 85% accuracy.	-Laboratory bacterial contamination -Microbiome shared by individuals (for example cohabiting couples and family members). -Stability of skin microbiomes collected over time intervals -Further body sites and markers of bacterial genus
Watanabe et al., 2018 [12]	-11 individuals, 66 samples for 2 years (33 for each year) -A public dataset (837 publicly available skin microbiome samples) of skin microbiome samples from 89 individuals	-Personal identification accuracy of 95% (63/66); using three reference samples from the first year and three query samples from the second year, they found accuracy of 85% (28/33). -Using a public dataset (89 individuals), they calculated a personal identification accuracy of 78% (663/837).	-The need of extensive dataset of sampled skin microbiome and of more studies on temporal dynamics of the microbiome
Park et al., 2017 [11]	-15 individuals, 696 bacterial strains isolated from palm hand	-They found personal variation of some minor species and hypothesized the major species could apply as molecular biological markers	-Sample size -Features of the individuals, without explaining how these factors could influenced the results (for example, taking of antibiotics).
Meadow et al., 2014 [15]	-51 samples from 17 participants (3 samples for each individual)	-They mainly found that an individual’s finger shared on average 5% more OTUs with his or her own phone than with everyone else’s phones (*p* < 0.001).	-Environmental conditions -Sample size -Design of study (teaching exercise) -Type of phone (only smartphones without keypads) -Hand-washing methods variable not considered
Neckovic et al., 2019 [13]	-6 individuals placed into 3 pairs for a total of 65 samples	-The Jaccard and unweighted Unifrac distances between samples, revealed that the clustering of participant pairs was distinct.	-The need of negative controls -Bacterial contaminations -Sample size
Lax et al., 2015 [14]	-2 individuals (total samples 315) -Geographically study: 89 individuals (unknown total number of samples)	-They assessed that phone-associated microbial communities were observed to be both less stable (higher median distance) and more variable in their rate of change over time (broader distribution) than shoe-associated communities.	-Rapid turnover of the surface-associated microbial community -Sample size -Short sampling time (two days) -Few substrates analyzed
Costello et al., 2009 [16]	-For the first study: 815 samples (7–9 adults on four occasions) -For the second study: *n* = 16	-Within habitats, interpersonal variability was high, while individuals exhibited minimal temporal variability. -Environmental characteristics play a strong role In shaping skin bacterial communities	-The need of a longer observation period to assess the influence of environmental factors and historical exposures
Phan et al., 2020 [17]	-45 individuals (left and right hand were obtained from each subjects: total of 90 samples	-They found Alloiococcus species could be a potential biomarker for sex (64% accuracy rate) and ethnicity (56% accuracy rate)	-Large standard deviation in samples Lack of clear distinction between groups -Low robustness of predictive models (apart from sex prediction) -Low prediction accuracy rates -Small sample size uneven breakdown of groups within each examined factor considering other substrates than playcards -Bias of individuals: same group (university) and not considering if they were co-habitants or if they had pet -Single site point: subsequent sampling
Richardson et al., 2019 [18]	-37 individuals	-They predicted the sex of the individual (error ratio of about 2.5, and accuracy of around 80%)	-Population of students living in the same dormitory -The bias of presence of roommates
Fierer et al., 2008 [19]	-51 individuals, 102 samples from 27 M and 25 W	-Hands from the same individual shared only 17% of their phylotypes, with different individuals sharing only 13%. This intraindividual differentiation was not significantly affected by handedness, sex, or hand hygiene (*P* < 0.05 in all cases). Men and women harbor significantly different bacterial communities on their hand surfaces (*P* < 0.001).	-Sample limited to students -The lack of detailed information on the skin characteristics of the sampled individuals.
Bell et al., 2018 [20]	-10 cardiac tissues	-sex-dependent changes in the thanatomicrobiome composition were statistically significant (*P* < 0.005).	-Small sample size -Analysis of the variability of the bacterial community based on the time elapsed since death -Other substrates into consideration
Tridico et al., 2014 [21]	-Forty-two pools of DNA extracts from 7 individuals (4 F and 3 M)	-Lactobacillus spp. was found in the female pubic hair samples and not in the male samples (excepting in the cohabiting male). Instead, similar microbial taxa were observed in the cohabiting couple, suggesting interindividual transfer. In contrast to the pubic hairs, scalp hair microbiota showed no correlation with the sex of the donor. Moreover, pubic hair microbiomes appeared to be less influenced by environmental bacteria than scalp hairs.	-Sample size -Scientific validation including: replication, persistence of bacteria during contact and stability during storage
Pechal et al., 2018 [22]	-83 individuals	-Decreased phylogenetic diversity was observed as a significant predictor (*P* = 0.038) of heart disease	-Age bias of individuals
Nagasawa et al., 2013 [23]	-10 individuals	-Phylogenetic tree of H. pylori showed 3 major clusters. All of the Japanese (*n* = 10), South Korean (*n* = 1), and Chinese (*n* = 2) cadavers examined in the present study were classified as type I, the single Thai cadaver was classified as type III, and the single Afghan and Filipino-Western cadavers were classified as type II. Different classification in this study could be due to external factors (i.e. latent origini)	-Results influenced by the latent origin -More geographical origins and knowing the background details of the analyzed sample, mostly unknown in this article
Escobar et al., 2014 [24]	-Total individuals 126, 30 Colombian adults	-The UniFrac analysis indicated that the gut microbiota of Colombians was significantly different from that of Americans, Europeans and Asians (*P* = 0.001). Moreover, they found that the relative abundance of Firmicutes decreased with latitude (*r* = − 0.27, *P* = 0.002) and that of Bacteroidetes increased with latitude (*r* = 0.28, *P* = 0.001)	-Sample size -No statistical power (no previous data on Cloumbians, high variability) -Influencing environmental factors (i.e. diet)
Brinkac et al., 2018 [25]	-Total individual of 21, for a total of 42 and 32 hair samples from scalp and pubis respectively.	-Scalp hair showed greater potential to predict geolocation than pubic hair	-Influencing factors: hair length, sebum content, lifestyle factors -Sample size -The need of longitudinal studies
Ghemrawi et al., 2021 [26]	-10 genital samples (10 individuals)	-They found that the penile microbiome species composition was different from vaginal	-Sample size -The need of longitudinal studies -The sample not included couples neither information regarding recent intercourse -Preliminary study
Williams et al., 2019 [27]	-43 individuals, 155 total sample collections	-A correlation between the proportion of couple co-clustering and the frequency of sex intercourse Increased frequency of sexual activity didn’t however guarantee increased microbiome similarity	-Larger sample sets -The need of controlled studies
Dixon et al., 2023 [28]	-6 male-female sexual partners pair, 20 swabs for couple	-Both the male and female genital microbiomes might be susceptible to alteration by the opposite sex	-More specific details (specific intimate behaviors, menstruation, health status, time of sampling) -Larger study group
Kennedy et al., 2012 [29]	-16 individuals	-The 16 S rRNA model revealed a sensitivity of 100%, with a 25% false positive rate. The ITS model found a 65% chance of obtaining a false positive. Finally, the rpoB model matched all bite marks to the corresponding teeth	-Sample size -Self-produced bite marks -Influencing factors (for example dental diseases) -Difficult adaptation to reality

According to the different aspects of identification, the results were categorized into the following four topics:

I. personal microbiome and transfer to the surrounding environment;.
II. microbiome as indicator of biological profiling features;
III. geolocalization;
IV. determination of sexual contact.

At the end of each article, the individual limits are discussed in detail, whereas the main limitations, common to the various studies analysed, are summarized and discussed in a separate section.

### Personal microbiome and transfer to the surrounding environment

According to the Locard’s Exchange principle which posits that *“every contact leaves a trace”*, human microbial communities have been studied to understand their role in binding an individual to the surrounding environment, as a *“personal microbial cloud”* [7].

Fierer et al. [8] conducted three studies to demonstrate the potential utility of human microbiome for forensic identification. In the first one, they compared bacterial communities on individual keys of three computer keyboards to the communities found on the fingers of the keyboard owners. In the second one, they linked objects to specific individuals by comparing the bacteria on their computer mice against a database containing bacterial community information for more than 250 hand surfaces, including the hand of the owner. Analyzing bacterial 16 S rRNA gene sequences, they found a degree of similarity between bacterial communities (represented in plots generated using the pairwise unweighted and weighted Unifrac distances) on fingertips of the three individuals sampled and their respective keyboards. They also demonstrated that the fingertips of an individual bacterial communities are more similar to those found on the keys of that individual’s keyboard than to those communities found on key-board keys not touched by the individual. In the last study, they aimed to determine whether bacteria on a personal object more closely resembled the owner’s skin bacteria than those of the general population. They calculated the phylogenetic distance between the bacterial communities on 9 personal computer mice and mouse owner’s hand, comparing it to the distances between the mouse bacterial communities and the communities on 270 hands that had never touched the mouse. They found that in nearly all cases the bacterial community on a given mouse was significantly more similar (using the unweighted and weighted Unifrac distances) to those on the owner’s hand than to the hands in the database. They found that there was a similarity between the microbiome present on personal items and the subject to which they belonged, suggesting direct transfer of bacteria from fingertips. In all nine cases, the bacterial community on each mouse was significantly similar to the community in the owner’s hand than in the other hands in the database.

The study also considered the effect of storage conditions on collected skin-associated bacterial communities, revealing that these conditions had little to no influence on bacterial community composition for up to 14 days. Regarding this point, laboratory conditions as typical of indoor environments (temperature at 20 °C and fluorescent lighting on for 8 h a day), although necessary for the study, differed significantly from the reality. Despite these conclusions, the sample size and the selection of individuals who worked within the same building (two individuals from the keyboard study shared the same office space) could represent limitation to the forensic application [8].

In their studies, Schmedes et al. [9, 10] firstly collected samples from 14 skin body sites from 12 healthy individuals sampled at three time points over a 2.5-year period. They identified stable clade-specific markers that provided individualizing resolution at each body site. It was based on skin microbiome profiles generated using nucleotide diversity (i.e., a measure of strain-level heterogeneity of the microbial population) of each marker. They used *Proprionibacterium acnes* pangenome presence/absence features and the nucleotide diversities of clade-specific markers to identify stable features which can be used to attribute skin microbiomes from multiple body sites to their respective hosts. The manubrium site and the hypothenar palm yield highly accurate rates of classification (97% and 96% respectively). Nucleotide diversity of stable markers reached accuracies as high as 100% from cheek, inguinal crease and popliteal fossa and contributed significantly greater to classification accuracies than presence/absence features (*p* < 0.01) [9].

They also developed a novel targeted sequencing model, the hidSkinPlaex, to attribute skin microbiomes collected from eight individuals from three body sites (i.e., foot, hand and manubrium) to their host donor. Three replicate samples were collected from each body site for a total of nine swabs collected per individual (*n* = 72). The panel consisted of 286 clade-specific markers from 22 bacterial with > 65% of the markers from P. acnes.

Skin microbiome profiles were assessed using subsets of universal (i.e., markers common to all individuals and body sites) and non-universal markers (i.e., all markers present across all samples). The comparison between these two categories showed an accuracy (i.e. the percentage of samples classified correctly) higher and statistically positive (*p* < 0.00001) using enriched hidSkinPlex markers from foot microbiome, as opposed to markers from shotgun data. Enrichment of hidSkinPlex markers provided the capability to identify skin microbiomes from individuals when the body site was unknown to the classifier with up to 97% accuracy using markers shared across the three body sites. It also gave the ability to identify the body site origin of the skin microbiome sample with up to 86% accuracy. Thus, the hidSkinPlex could serve a dual purpose, providing a method to not only identify individuals but also predict the body site origin of skin microbiome samples [10]. These studies have highlighted these principal limitations, also reported by the authors: the laboratory bacterial contamination, the sharing of microbial communities between individuals (for example cohabiting couples and family members), the need to analyze further markers of bacterial genus and the stability of skin microbiomes collected over time intervals, the latter not analyzed in this study.

Park et al. [11] collected samples from 15 individuals (right-handed and healthy, 4 smoking and one who had taken an antibiotic), exploring microbial communities inhabiting their palms obtained by hand-printing and using culture-based methods. A total of 686 bacterial strains were isolated (only with aerobic cultivation) and identified based on 16 S rRNA gene sequence analysis. The genus *Staphylococcus* was detected in all participants, and *Micrococcus* and *Enhydrobacter* were detected in most participants (87% and 80% of the cases, respectively). Despite the small number of the sample, some minor species were unique for specific individuals. They concluded explaining that some major species could also applied as molecular biological markers at subspecies level and minor species could be potential used to human identification. The sample size and the inclusion of individuals with characteristics that could have influenced the results represent major limitations. For example, smoke and taking antibiotics were not explored by the authors [11].

Watanabe et al. [12] investigated the contribution of minor skin taxa to the effectiveness of personal identification, selecting the forehead microbiome as a skin microbiome model, due to the presumed minor contact of this part of the body with objects or other individuals (considering some skin parameters as moisture, pH and sebum). They recruited 11 individuals (original dataset) and collected 66 forehead microbiome samples at six different time points over two years (33 samples each year). To assess the microbial taxonomic composition of each sample, the 16 S rDNA were PCR amplified. They calculated the Canberra distance between a query sample (unknown individuals) and reference samples (known individuals). They evaluated a personal identification accuracy of 95% (63/66). Moreover, they tested the accuracy acquiring data in different years. Using 3 reference samples from the first year and 3 query samples from the second year, they found the accuracy to be 85% (28/33). Furthermore, they evaluated the method using a public dataset (89 individuals) and calculated a personal identification accuracy of 78% (663/837), noting that the accuracy of personal identification increased with higher reference samples per individuals. In this study authors revealed that the taxonomic composition of the skin microbiome was mostly stable over a short period (i.e. up to a few months) but fluctuated slightly over extended periods (i.e. >1 year), suggesting that the intra-individual taxonomic composition of the human skin microbial community was relatively stable.

Despite these promising results, the stability of the microbiome should be studied over longer periods of time, using a larger number of individuals and testing other body parts, considering all specific influencing factors. In fact, this is one of the few studies that uses the forehead as a source of microbiome, which has been hypothesized to be less influenced by external contact (e.g. sebum production). On the contrary, more studies on larger populations should verify the influence of other factors on bacterial communities (including those proposed to the authors themselves) [12].

Neckovic et al. [13] considered the potential for human skin microbiomes to be transferred between non-cohabitating individuals, and from an individual to substrates, through direct and indirect contact. They involved six participants placed into three pairs, taking part in direct and indirect modes of transfer. The first mode was measured through the act of a handshake with another individual, followed by contact with a substrate. The second mode involved individuals rubbing a substrate in their left hand, swapping substrates with their partner and then rubbing the swapped substrate in their left hand. A total of 65 samples underwent 16 S rRNA sequencing. The Jaccard distances (a proximity measurement used to compute the similarity between two objects: a value of 0 is indicated as the distance between a sample and itself, whereas a value closer to 1 would indicate a greater distance and therefore, less similarity in microbial community composition) between the reference samples of each participant were all greater than 0.8, meaning there was dissimilarity in the microbial compositions of the skin microbiomes between participants. Each individual reference sample was observed to cluster either within or around the samples of each respective pair, exhibiting closer distances to their mixed samples than to those belonging to another participant pair. The statistical results, illustrated in plots and based on Jaccard and unweighted UniFrac distances between samples, revealed distinct clustering of participant pairs. This suggested that, following direct or indirect transfer of hand-associated microbiomes, this form of analysis may be used to associate individuals with other individuals and/or substrates. The forensic application of the results could be hindered by some elements, first of all, the short sampling time (within three days) which does not allow the transitions or variation in microbial to be appropriately assessed. Furthermore, several factors (such as the relative surface areas contacting each other, the level of pressure and friction applied during the contact, and the duration of the contact) that may influence the microbiome detected on hands and skin should be taken into consideration. Finally, for the purposes of applicability to the real context, contamination risks associated with all people or objects that came into direct contact with the skin/body site in a specified period and with the type of interaction should be considered. These results should also be integrated with the introduction of negative controls, i.e. free from contaminating microbial DNA [13].

Lax et al. [14] recruited two participants to sample their phones, soles of their shoes and the floor over the course of two 12-hour time periods on two consecutive days. A further 89 participants took individual samples of their shoes and phones at three different scientific conferences. Random forest models were used to determine which of the two individuals’ shoes a sample was taken from, correctly classifying samples more than 50 times as effectively as one would expect by chance. In phone samples, the models were able to classify the participant a phone sample was taken from (error ratio of 13.6). Random forest models were able to determine which of the three conferences a sample was taken from significantly better than expected by chance for both the shoe and phone environments (error ratio = 11.7 and 8.0, respectively).

Regarding the stability of microbial community, they analyzed the dissimilarity in community composition and considered the phylogenetic distance. They assessed that phone-associated microbial communities were observed to be both less stable (higher median distance) and more variable in their rate of change over time (broader distribution) than shoe-associated communities. They hypothesized that the high volatility of phone-associated microbial communities was likely due to a small microbial biomass that would be prone to a rapid turnover in community composition and to the very high volatility of hand-associated microbiota [14].

They showed temporal variability in the differentiation in the shoe microbial communities of these two different people. In contrast, the models were unable to determine the specific site where the sample had been taken (for all substrates analyzed). They hypothesized that this was due to the homogenization of communities across the shoe sole over time or to rapid changes in community structure at each sampling site.

This study suggests how the microbiome can be used to trace objects to their owners and to lead an individual back to a place. The short sampling time (two days), the small sample and the few substrates analyzed (telephone, floor and sole) represent the major limitations. Furthermore, it should be explored the surface-associated microbial community and whether shoe sole material and turnover could influence bacterial communities.

Meadow et al. [15] characterized microbial communities on seventeen individuals’ smartphone touchscreens sampled from the touch-surfaces of their own mobile phone, as well as their own thumb and index finger on their dominant hand (3 samples for each of 17 participants, with a total of 51 samples). They found that the two fingers from each participant had significantly more in common than either did with phones (*p* < 0.001 for both fingers). Handwashing made an insignificant difference in the resemblance of the two fingers (*p* = 0.126) and in the finger/phone connection (*p* = 0.7). Women’s fingers appeared to share more operational taxonomic units (OTUs) with their phones than men, but the difference was not significant (*p* = 0.128), since both shared more OTUs on average, with their own phones than with anyone else’s. Indeed, they found that an individual’s finger shared on average 5% more OTUs with his or her own phone than with everyone else’s phones (*p* < 0.001). The author explained several limitations of their study: sample size, the design of the study as a teaching exercise, the lack of information about the environmental process of breeding microbes on phone’s touchscreen and the factors that could influence it (e.g., material type, temperature, pH, humidity, exposure to ultraviolet light and substrate availability). Furthermore, the authors only considered mobile phones equipped with touchscreens (smartphones) and not those equipped with a keyboard, neither they distinguished hand washing methods that could also influence the results [15].

Costello et al. [16] conducted a study on the spatial and temporal distribution of the human microbiota, surveying bacteria from up to 27 sites in 7–9 adults on four occasions. They collected 815 samples and found each habitat harboring a characteristic microbiota and a relative stable set of abundant taxa across people and over time. They assessed differences in overall bacterial community composition using UniFrac metric (a small distance implies that two communities are similar). For each sample, variable region 2 (V2) of the bacterial 16 S rRNA gene was PCR-amplified. They detected a characteristic microbiota for each habitat and a relatively stable set of abundant taxa across people and over time. Indeed, they found that composition varied significantly less within habitats than between habitats. Within habitats, variation was significantly less within individuals sampled over time than between individuals on a given day. After accounting for habitat and host individual, variation was significantly less over 24 h than over 3 months (*p* < 0.01). Despite the strong inter- and intrapersonal structuring of bacterial diversity, a high degree of spatial and temporal variability was also evident: about 12% of phylotypes appeared on all dates, while 3% of phylotypes appeared in all individuals, and only 0.1% of phylotypes appeared in all body habitats [16]. Despite these results, a longer observation period and studies on influencing factors like local chemistry and nutrient availability are needed. For example, the forehead has been identified as a more susceptible site to external factors (mainly the production of sebum).

### Microbiome as indicator of biological profiling features

Phan et al. [17] investigated how the bacterial profile could be used as an indicator of donor characteristics such as sex and ethnicity. In their study, forty-five individuals were asked to hold an autoclave-sterilised playing card, which was subsequently swabbed and the samples collected over the course of two weeks. The difference in microbiota diversity was examined using weighted (quantitative assessment) and unweighted (qualitative assessment) UniFrac distances. They found *Alloiococcus* species could be a potential biomarker for sex (64% accuracy rate, indicating male donor) and ethnicity (56% accuracy rate, indicating donors of Caucasian and mixed ethnicities). In addition, other characteristics (including diet and use of hand sanitizer) were also investigated. Analysis showed *Lactococcus* as a marker for Chinese diet type with a 48% prediction accuracy rate. Finally, concerning the use of hand sanitisers, *Alloiococcus* was present in only 43% of the bacterial traces from donors who used hand sanitisers compared to being present in 72% of the bacterial traces from donors who did not use hand sanitizers, with a 51% accuracy rate (*p* = 0.003 for unweighted Unifrac distances of microbial community) [17].

The limitations highlighted in this article were the sample size, the large standard deviation in samples, the bias of subjects introduced (all university students) and the low robustness of the predictive models for most features tested, such as sex. Furthermore, in this study no information was noted about the history of contact of the subjects with other objects or the presence of cohabitants or coexistence with pets. More in-depth analyzes could also reveal similar results using a different substrate. Finally, as this study examined a single time point, it is unknown whether any of the identified bacteria would remain or be in similar abundance in subsequent sampling. Expanding the sample size, diversity of the subjects and temporal scope would yield a greater wealth of information on the potential links between microbial signatures and donor characteristics of forensic interest [17].

Regarding sex, Richardson et al. [18] collected personal samples from the hands and other objects in the room of 37 students living in a common dormitory, distributed across 28 distinct dorm rooms. Through the study of specific microbial taxa, they identified the sex of the subject with the DESeq2, a statistical method for differential analysis of count data, using shrinkage estimation for scatters and fold changes to improve stability and interpretation of estimates. Examining *Lactobacillus* and *Corynebacteria* species, the random forest model was able to predict whether a subject was male or female, with an error ratio of about 2.5, and accuracy of around 80% on the test set. The major limitation of this study was the presence of roommates, since interactions between individuals involve an exchange of bacterial communities and therefore a decrease of differences in taxon abundance. In this study, an individual’s classification error was linearly related to the number of roommates that individual had, with classification error increasing by 18% points for each additional roommate [18].

Fierer et al. [19] collected samples from the palmar surfaces of both the hands of 51 students to characterize bacterial diversity on hands and to assess its variability within and between individuals. They observed intra- and interpersonal variation in bacterial community composition: hands from the same individual shared only 17% of their phylotypes, with different individuals sharing only 13%. This intraindividual differentiation between the bacterial communities on left and right hands was not significantly affected by handedness, sex, or hand hygiene (*p* < 0.05 in all cases). Men and women harbor significantly different bacterial communities on their hand surfaces (*p* < 0.001). In this article the limitations could be the presence of a sample restricted to a population of students and the lack of detailed information on the skin characteristics of the sampled individuals. So, it could be difficult to understand whether sex differences in bacterial communities on the hands may be due to skin factors, for example pH, sweat or sebum production, frequency of moisturizer or cosmetics application, skin thickness or hormone production [19].

Bell et al. [20] examined thanatomicrobiome (i.e., postmortem microbiome) by collecting heart samples from 10 individuals who died of sudden cardiac arrest with times of death ranging from 6 to 56 h. They amplified the V1-V2 and V4 hypervariable regions of prokaryotic 16 S RNA genes. Individual OTUs were examined and the relative abundances of the most abundant microbial taxa in all samples relative to region (V1-2 and V4) were determined. Their study revealed a distinction in the heart thanatomicrobiome of male and female corpses at all taxonomic levels. For example, at the order level, *Lactobacillales* and *Rhizobiales* were only detected in males and *Pseudomondales* in females. Their results showed that sex-dependent changes in the thanatomicrobiome composition were statistically significant (*p* < 0.005). In this study, apart from the small sample size, the major limitation is due to the lack of in-depth analysis of the variability of the bacterial community based on the time elapsed since death. Furthermore, because of the only substrate used was the heart, these results should be validated taking other substrates into consideration [20].

Tridico et al. [21] surveyed bacterial communities, associated with human scalp and pubic hair, from seven healthy Caucasian individuals of both sexes (two of whom were in a relationship), ranging in age from 23 to 53 years old. Samples were collected at three time points, initial collection in addition to 2 and 5 months thereafter. Forty-two pools of DNA extracts were obtained from human scalp and pubic hairs. Data generated from pubic hair held (using next generation sequencing) revealed a dichotomy between OTUs on male and female pubic hair shafts. *Lactobacillus spp.* was found in the female pubic hair samples and not in the male samples (excepting in the co-habiting male). Instead, similar microbial taxa were observed in the cohabiting couple, suggesting interindividual transfer, especially after sexual intercourse. In contrast to the pubic hairs, scalp hair microbiota showed no correlation with the sex of the donor. Moreover, pubic hair microbiomes appeared to be less influenced by environmental bacteria than scalp hair [21]. The temporal stability study found that pubic hair bacteria may be more temporally stable than scalp hair bacteria and therefore potentially of greater evidentiary value than scalp hair bacteria. Data showed that about 17% of pubic hair bacterial OTUs were temporally stable at all time points; while, on average, scalp hair hosted approximately 5% bacterial OTUs. Despite these findings, more studies should be conducted on the role of bacterial transfer during contact, the temporal persistence of bacteria after transfer and sample storage conditions. In fact, the forensic application could also be useful in cases of suspected sexual violence. The temporal persistence of the bacterial community on the pubic hair should be studied, especially since the examination on the victim is often not carried out acutely.

Pechal et al. [22] studied the thanatomicrobiome as a sign antemortem health condition, which could be used to complement the biological profile. They analyzed microbial taxonomic profiles from a total of 83 cases (less than 24 h postmortem), divided into two groups: cases with evidence of heart disease detected during autopsy and cases with death resulting from violent. Heart disease was based on examination of the heart (including microscopic analysis) and medical history. To assess whether there were statistical associations between the postmortem microbiome and antemortem health status, they ran binomial logistic regression models to contrast community diversity with heart disease. They examined the bacterial community from the mouth, finding phylogenetic diversity in cases of heart disease (with significant predictive factor, *P* = 0.038). In contrast, individuals whose death was due to violent circumstances had greater microbial diversity. These data suggested that increased microbial biodiversity may be an indicator of individuals without chronic health conditions, such as heart disease. This study could be biased by the age of the subjects included (in the original dataset, 44 ± 15 years), as heart diseases typically appear later in life and are chronic conditions, whereas violent deaths tend to involve younger individuals. Studies evaluating the bacterial community at multiple collection times (for example, near, at and after death) should be conducted [22].

### Geolocation

In 2010 the Earth Microbiome Project (EMP) was founded^1^. It represents a systematic attempt to characterize global microbial taxonomy with the aim of understanding biogeographical variations and the factors, such as climate, altitude, latitude, or soil nature, that determine them. Indeed, the characterization of the microbiome may provide information on the geographical origin of the individual.

In their study, Nagasawa et al. [23] developed a method to determine the geographic origin of 17 cadavers with known geographic origins by examining the H. pylori vacA region polymorphism. VacA is a cytotoxin that comprises two variable parts in the region of the vacA gene: the s-region (s1 and s2) and the m-region (m1 and m2). East Asian H. pylori strains are associated with the vacA s1 type; within East Asian countries, the m1 type predominates in Japan and Korea, whereas the prevalence of the m2 type gradually increases in the southern parts of East Asia. Phylogenetic tree of *H. pylori* showed 3 major clusters consisting of the East Asian type I, including Japan, China and South Korea, the Western type II, including Russia, the Americas and Europe, and the Southeast Asia type III, including Thailand, Hong Kong, Taiwan, and Vietnam. All the Japanese (*n* = 10), South Korean (*n* = 1), and Chinese (*n* = 2) cadavers examined in the present study were classified as type I, the single Thai cadaver was classified as type III, and the single Afghan and Filipino-Western cadavers were classified as type II. Even if Filipinos and Taiwanese are typically classified in the type III cluster, different classification in this study could be due to external factors. In fact, the Taiwanese cadaver was classified as type I, probably since the individual was recorded as being an ethnic Taiwanese, but had lived in Japan from childhood. These findings demonstrate the influence of geographic and latent origin of the cadavers on this method. These considerations recall the difference between geographical origin and ethnicity, which is still provided by the analysis of the polymorphism of the human genome. More studies should be conducted including more geographical origins and knowing the background details of the analyzed sample, mostly unknown in this article [23].

Escobar et al. [24] described the composition of the gut microbiota comparing Colombian adults with different geographic origin (USA, Europe, Japan and South Korea). They included a total of 126 individuals, of which 30 were Colombian. Each participant collected a fecal sample. They found that the gut microbiota of Colombians was mostly composed of Firmicutes (average ± SD: 79 ± 13%) and Bacteroidetes (17 ± 12%), followed by other phyla present in minor frequencies. The remaining datasets had lower proportions of Firmicutes and higher proportions of Bacteroidetes but dispersion of data among individuals was equally notorious than in the Colombian dataset. The UniFrac analysis indicated that the gut microbiota of Colombians was significantly different from that of Americans, Europeans, and Asians (*p* = 0.001). Moreover, they found that the relative abundance of Firmicutes decreased with latitude (*p* = 0.002) and that of Bacteroidetes increased with latitude (*p* = 0.001). The authors highlighted that the sample size was not designed to achieve statistical power due to the lack of previous data on Colombians and the highly variable results of studies performed on other populations. Moreover, due to the influence between geographic origin and diet, they concluded that it would be interesting to tease apart the effect of diet and geography on the composition of the gut microbiota [24].

Brinkac et al. [25] conducted a study comparing the variation in scalp and pubic hair microbiome across different geographic origins. They collected hair samples derived from scalp and pubic areas from adults residing in Maryland (MD, *n* = 8) and California (CA, *n* = 8). Additionally, scalp hairs were collected from adults residing in Virginia (VA, *n* = 5). Each individual provided multiple samples for a total of 42 and 32 hair samples from scalp and pubis respectively. They observed that the *Peptoniphilus* and *Staphylococcus* genera had different sample abundances between MD and CA, with no significant clustering by geographic location in each hair type. Compared to hair, the analysis of pubic hair revealed a higher error rate (22.58% compared to 17.24% for hair), suggesting that scalp hair had greater geolocation prediction power than pubic hair. More studies should be included to understand hair characteristics that may influence these results: for example, length, hair collection technique (cut or plucked), sebum production, environmental or lifestyle factors. Increasing sample sizes and performing longitudinal studies would help further clarify the usefulness of both scalp and pubic hair as indicators of forensic information [25].

### Determination of sexual contact

The human microbiome has been hypothesized to be potentially useful in studies investigating its transfer during sexual contact. Ghemrawi et al. [26] described genital microbial signatures based on the analysis of five male and five female genital samples (for a total of 10 samples) and compared these results to those from longitudinal studies. They did not include couples in the study, and no information was collected regarding recent intercourse. The shotgun sequencing results showed taxonomic diversity and richness of the penile microbiome, as opposed to the vaginal ones which were composed predominantly of lactobacilli (about 76% of the total vaginal composition). The authors classified this study as a “pilot study,” which should be complemented with a larger sample and longitudinal studies. In fact, some factors that could have influenced the results should be connected: on collection time, the absence of information on previous sexual intercourse and the presence of other variables (for example circumcision or the day of the menstrual cycle on which the sample was taken) [26].

Williams et al. [27] collected microbiome profiles from pubic hairs and/or swabs taken from the pubic mound region of 43 participants (including 12 partner pairs). Participants provided 1 to 5 sets of sample collections (in 3 set time points) resulting in 155 completed sample collections. Individuals were stratified based on many characteristics, such as sex, age, ethnicity, sexual activity, condom use, and oral to genital contact. Results showed that the two couples who did not report sex in the seven days prior to sample collection for any of the time points were the only couples whose male and female samples consistently fell into separate clusters About the influence of the level of sexual activity, they found a significant correlation between the proportion of couple co- clustering and the average number of times the couple reported having sex during the seven days preceding each sample collection. Increased frequency of sexual activity didn’t however guarantee increased microbiome similarity (for instance, two couples were similarly sexually active but clustered together 33% and 80% of the time, respectively). This result established that sexual activity per se was not sufficient to ensure microbiome sample sharing and made it unlikely that a single incidence of intercourse could always result in detectable transfer. This study would require a larger sample size and greater control over some variables. For example, we do not know whether voluntary sexual contact may have different characteristics than that conducted by force. Furthermore, controlled studies involving collection of samples immediately prior to sexual contact and then at fixed time points after it would serve to quantify the variability in proportion of transfer both to hairs and the pubic mound, and for how long any mixing is retained [27].

Dixon et al. [28] studied the variation of bacterial communities in six male-female sexual partner pairs before and after sexual intercourse, also controlling for female cyclic variation and selecting strict parameters to simulate a single episode of penetrative sexual encounter. Five replicate swabs (penil skin and vaginal) were collected for each participant and timepoint, totaling 20 per couple. (10 male, 10 female). Taxonomic analysis found that in both male and female samples, there was an increase in the total genera observed post-coitus. The most notable change in abundance postcoitus was the increase in male samples of the dominant female taxa, Lactobacillus. Few changes were observed in female. In three female samples, an increase in the distance between the samples was observed before and after coitus, while the male samples observed a progressive clustering after coitus. In contrast, in a pair, female before-and-after samples are tightly clustered, while male samples have a larger distance between each other. The authors hypothesized that both the male and female genital microbiomes might be susceptible to alteration by the opposite sex. Despite these results, the authors highlighted some limitations. They did not know what specific intimate behaviors occurred during their sexual encounter, making it difficult to hypothesize a relationship between microbial diversity and intercourse effect. Therefore, larger study groups should analyze circumcision as a penile skin variable and evaluate additional time points to assess microbiome recovery. Finally, they did not consider that the partners could be sexually active during menstruation, and it is also conceivable that the volunteers did not respect the abstinence period. They should also collect more information on people’s health and the time of sampling, in order to reduce accidental factors or contamination [28].

Since bite mark injuries could be present in sexual abuse, Kennedy et al. [29] assessed the matching oral streptococcal DNA sequences from bite marks to those obtained from the teeth responsible. They also evaluate the capability of three genomic regions of streptococcal DNA to discriminate between participant samples. They enrolled 16 individuals who generated self-inflicted bites on their upper arms. The following genetic targets were examined: the hypervariable regional 9 of streptococcal 16 S rRNA gene, a stretch of noncoding DNA located between the 16 S and 23 S rRNA genes (ITS), and a stretch encoding the beta subunit of bacterial RNA polymerase (rpoB). The 16 S rRNA model revealed a sensitivity of 100%, with a 25% false positive rate. The ITS model found a 65% chance of obtaining a false positive. Finally, the rpoB model matched all bite marks to the corresponding teeth. Upon achieving perfect discrimination, they demonstrated the complete ability to differentiate between samples from teeth responsible for a bite and those not responsible.

A major limitation of this study, besides the sample size, is that the bite marks were self-inflicted, and it did not analyze how diseases affecting dental elements, such as cavities, could lead to microbiome variability. Furthermore, adapting this study to reality, it is not known how long the microbiome left by the bite can survive temporarily and whether it can be influenced by a microbiome not only from a different site of the body, but from another individual [29].

### Limitations

For the human microbiome to be effectively applied to identification in forensic science, it must exhibit temporal stability and specificity to particular body sites and to sex. Furthermore, the mechanisms that involve the transfer should be explored in depth, so that the variables that may influence the changes can be predicted. These variables are divided into environmental factors, lifestyle choices, and internal factors, which also include the subject’s state of health. In the selected studies, specific limitations were identified and described. Furthermore, all studies share the following limitations:

- the instability of the microbiome to intrinsic and extrinsic factors, for example the use of antibiotics or the presence within the subject of a certain disease or hormonal factors that modify the microbiome;
- the difficulties in maintaining the ideal conditions in carrying out the sampling, transport, and treatment of the microbial community: in fact, different microbial populations may require different protocols. Furthermore, according to what is accepted by the scientific community, a valid protocol should have been tested in field conditions, whether it has been subjected to peer review, whether the rate of error is known, standardization and whether it has been generally accepted. This scientific methodology is the way to ensure the reproducibility and comparability of research results to be applied in concrete cases of judicial investigation (with the same safeguards in terms of privacy and confidentiality as any other human tissue samples or identifying sources of information);
- the presence of contaminants of human, environmental and other living beings, such as animals and insects;
- the sample size of the studies presented in this review, which appears to be still too small;
- the standardized definition of changes in the microbial community in the postmortem period. The postmortem human microbiome, such as Javan et al. reported, includes two components: the thanatomicrobiome, consisting of microbes that inhabit internal organs and body fluids after death, and epinecrotic microbial communities, represented by microbes found on the surface of decaying remains [4]. In fact, the thanatomicrobiome is conditioned by many endogenous and exogenous factors, including climatic conditions and the presence of animals, as well as by postmortem translocation and agonal diffusion phenomena;
- almost all of the studies presented (with the exception of the study by Kodama et al. [30]) were constructed according to a rigid design to control interfering variables (e.g., hand washing in subjects, voluntary recruitment of subjects). Nonetheless, such a study design could be difficult to adapt to real forensic applications;
- many studies should be conducted to identify a common matrix (i.e. a sampling site) less influenced by external and internal factors. For example, hands represent a useful site because they are more involved in contacts, but also more susceptible to confounding factors. On the contrary, for example the forehead is less susceptible to external contacts but influenced by individual factors (for example sebum production);
- robust information on the stability of the microbiome over time is lacking. Some studies included in this review explore these differences by including some time set points. However, information on baseline time and long term is often lacking.

Furthermore, it remains unclear how long microbial fingerprints can last unchanged on skin and surfaces, making it possible to analyze these traces and obtain reliable results. It was demonstrated, in fact, that skin microbiota shed by an individual can change over time, undergoing degradation within hours. A temporal variation was observed in human skin microbial composition. These factors can constitute a great limit to microbiome-based methods of human identification.

Kodama et al. [30] conducted a study where postmortem skin microbiomes and microbiomes from hand-held objects (e.g. phones, doorknobs) were collected to trace the associations between individuals and objects. In 16 death scenes, they swabbed the right palm of the decedent and personal objects at different times: on the scene of death, upon arrival at the morgue, and at 6-hour intervals thereafter until autopsy or external examination. A total of 98 objects were swabbed at the 16 death scenes, 88 of which yielded sufficient genetic material for sequencing. Postmortem skin microbiomes correctly associated with objects at an average accuracy rate of 75%, but the level of accuracy varied by scene. The observed variation was explained due to the time elapsed since the object was last touched, handling by other individuals and the nature of the objects, that could inhibit microbial colonization (e.g. cleansers, lubricants, and heat) [30].

Regarding methodology of this review, the lack of consistency and the heterogeneity of the studies, as well as the outlined limitations, preclude the performance of a meta-analysis. Furthermore, performing a quality assessment of the included studies was not feasible due to the vast range of study methodologies and the broad spectrum of definitions.

## Conclusions

Since it is not always possible to achieve forensic identification based on traditional sciences [31, 32], the microbiome has recently been studied as an alternative method. Despite the recognition of a potential use, there are still many limitations that do not allow us to reach a degree of probability of identification useful for establishing evidence, especially at Court. Even if there are few protocols for postmortem procedures by the experts in the field [33, 34], there is a lack of knowledge and sharing at territorial level and too much disparity among the various ways of operating in forensics, as for other field of forensic sciences [35, 36]. Today, forensic microbiology could serve as a supplementary tool, combined with traditional techniques, to potentially reveal more information about the individual in question [36]. The creation of a forensic microbiome “biobank” could facilitate the improvement of technologies for isolating and analyzing bacterial organisms, the development of a set of reference microbial genome sequences, the provision of new computational analysis tools for organisms, and the advancement of sequencing technologies.

## Data Availability

The datasets generated during and/or analyzed during the current study are available from the corresponding author on reasonable request.

## Footnotes

1 https://earthmicrobiome.org/.
